# Nomograms for postoperative complications in congenital biliary dilatation: a retrospective cohort study

**DOI:** 10.3389/fped.2025.1654592

**Published:** 2025-10-28

**Authors:** Yu Zhou, Xintao Zhang, Xue Ren, Dong Sun, Jian Wang, Aiwu Li

**Affiliations:** ^1^ Department of Pediatric Surgery, Qilu Hospital of Shandong University, Jinan, Shandong, China; ^2^ Department of Pediatric Surgery, Children’s Hospital of Nanjing Medical University, Nanjing, Jiangsu, China

**Keywords:** congenital biliary dilatation choledochal cyst, complications, machine learning, cholangitis, pancreatitis

## Abstract

**Objective:**

Postoperative complications after surgery for congenital biliary dilatation (CBD) can be life-threatening and often necessitate redo surgery. We aimed to predict postoperative complications in patients with CBD using machine learning (ML) algorithms.

**Study design:**

Data from pediatric patients with CBD who were surgically treated at our hospital between July 2014 and July 2023 was retrospectively analyzed. Multiple logistic regression and lasso regression were used to screen risk factors. Predictive models were developed using seven ML algorithms and the better-performing model was selected.

**Results:**

A total of 211 patients were included in the final analysis. Among these, 31 patients experienced complications (cholangitis: 14 patients; pancreatitis: 21 patients).Risk factors for complications identified by variable screening were preoperative perforation, Todani classification type IV-A (type 4A), days of removal of drainage (removal drainage), and serum amylase. Predictors of postoperative cholangitis were preoperative perforation, preoperative cholangitis, type 4A, removal drainage, anemia, level of serum albumin and amylase. Preoperative perforation, cholangitis, serum gamma-glutamyl transferase and amylase were predictors of postoperative pancreatitis. Finally, logistic regression was selected to develop the clinical prediction model for postoperative complications, cholangitis, and pancreatitis.

**Conclusions:**

We developed nomograms to predict postoperative complications, cholangitis, and pancreatitis after surgery for CBD using ML.

## 1 Introduction

Congenital biliary dilatation (CBD), also known as choledochal cyst, is a rare developmental malformation of the biliary system with a higher incidence in females (approximately male–female ratio 1:3) (1, 2). The incidence of CBD is highest in Asia (approximately 1/1,000 in Japan, and 0.3% in South Korea). In contrast, the incidence in western countries is much lower (one in 50,000–150,000) (3). Approximately 2/3 of patients with CBD are detected during childhood (4).

The typical clinical manifestations of CBD are abdominal pain, jaundice, and abdominal mass. Inadequate treatment can lead to liver function impairment, malnutrition, pancreatitis, bile duct perforation, and even cancer (5, 6). The first-choice treatment of CBD is complete excision with Roux-en-Y hepaticojejunostomy. With the recent advances in minimally invasive techniques, laparoscopic and robotic treatment are increasingly being used in CBD surgery.

Irrespective of the surgical approach (open or laparoscopic surgery), patients with CBD may develop postoperative complications such as anastomotic fistula, anastomotic stenosis, cholangitis, and pancreatitis. These complications can even be life-threatening (7), CBD-related complications are a concern of much debate (8, 9). However, there are no clinical prediction models for postoperative complications of CBD.

In this study, we discussed the risk factors for complications associated with laparoscopic surgical treatment of pediatric CBD. We used machine learning (ML) algorithms to construct a prediction model and develop a nomogram to provide a basis for preventing complications associated with CBD.

## 2 Material and methods

This study was approved by the Hospital ethical review committee. The presentation of this work follows the STROBE (Strengthening the Reporting of Observational studies in Epidemiology) criteria (10).

### 2.1 Study population

This was a single-center, retrospective cohort study. Clinical data, test parameters, and demographic data of pediatric patients (age < 18 years) with CBD treated between July 2014 and July 2023 were extracted from the electronic medical records. The inclusion criteria were as follows: 1) CBD diagnosed by preoperative imaging and clinical symptoms; 2) patients who underwent surgical treatment at our hospital and the diagnosis of CBD confirmed during the surgical procedure; 3) regular follow-up for more than one year. The exclusion criteria were as follows: 1) patients who underwent other procedures simultaneously; 2) patients with concomitant severe liver, kidney, lung, or other diseases; 3) incomplete data.

### 2.2 Definitions

CBD types: CBD was classified using the Todani classification of the Alonzo-Lej classification system (11).

Preoperative cholangitis (Pre-cholangitis): cholangitis was confirmed by postoperative pathology.

Postoperative cholangitis (Post-cholangitis): the presence of clinical symptoms of abdominal pain, jaundice, fever, and laboratory tests confirming abnormal liver function postoperatively.

Postoperative pancreatitis (Post-pancreatitis): No pancreatitis or pancreatitis was controlled preoperatively but pancreatitis developed postoperatively.

### 2.3 Data collection and outcomes

Data regarding the patient factors [sex, age, weight, anemia, preoperative perforation, preoperative cholangitis, whether the type of CBD was type 4A (11), and whether the shape of the cyst was cystic], surgical and clinical information (duration of surgery, intraoperative blood loss, intraoperative blood transfusion, duration of hospital stay, postoperative hospital stay, and removal of drainage), and pre-operative laboratory results and radiological data [cyst diameter, white blood cell count (WBC), platelet count (PLT), aspartate aminotransferase (AST), alanine aminotransferase (ALT), gamma-glutamyl transferase (GGT), AST to Platelet Ratio Index (APRI), alkaline phosphatase (AKP), total bilirubin (TBIL), direct bilirubin (DBIL), total protein, albumin, amylase, and lipase] were extracted from the electronic medical records.

### 2.4 Machine learning model building

#### 2.4.1 Selection of risk factors

Variables were screened by the least absolute shrinkage and selection operator (LASSO) regression analysis with 10-fold cross-validation and multivariate logistic regression. Predictive models were constructed for each of the screened variables. The covariance of all explanatory variables was assessed using a correlation matrix. Possible interaction terms were tested, revealing no significant interactions.

#### 2.4.2 Pre-processing of data

To maintain data integrity, factors with a substantial proportion of missing values (>20%) were excluded from the analysis. For variables with missing data, imputation was performed using the mean or median value, depending on the data type. The number of positive events was increased by a factor of five due to the low incidence of postoperative cholangitis and pancreatitis. The dataset was split into a training set (70%) and a test set (30%). Additionally, the data were standardized to ensure consistency in scale and range.

#### 2.4.3 Model selection

Logistic regression (LR), Support Vector Machine (SVM), K-nearest neighbours (KNN), Random Forest (RF), Extreme Gradient Boosting (XGB), Classification and Regression Tree (CART), and Neural Network (NN) were used to develop the prediction models respectively. All the developed models were compared using performance metrics including specificity, sensitivity (recall), accuracy, precision, receiver operating characteristics (ROC) and F1 statistics. Finally, the models with better performance were selected.

### 2.5 Statistical analyses

The SPSS 26.0 (IBM SPSS Statistics 26.0) and R software programs were used for statistical analysis. The normality of the distribution of continuous variables was assessed using the Shapiro–Wilk test. Normally distributed variables were compared using the *t*-test while the rank sum test was used for skewed variables. *χ*^2^ test was used to compare categorical variables. Univariate and multivariate logistic analyses were performed to identify variables affecting complications. ML algorithms were applied to determine the best predictive model. Statistical significance was defined as *P* < 0.05.

## 3 Results

### 3.1 Characteristics of the study population

A total of 211 patients (54 male; mean age: 3.39 years) were included in the final analysis. Of these, 31 patients experienced complications (cholangitis: 14 patients; pancreatitis: 21 patients). Besides, 4 patients developed calculus, 3 patients presented with intestinal obstruction, and 1 patient developed an anastomotic fistula. All the complications were resolved after treatment. Type-4A CBD was present in 59 patients. We randomly assigned the patients to the training and validation sets in a ratio of 7:3. The characteristics are summarized in Table 1.

**Table 1:** Baseline characteristics of included patients stratified by complication, cholangitis and pancreatitis.

Total (*n* = 211)	Non-complication (*n* = 180)	Complication (*n* = 31)	*P*	Non-cholangitis (*n* = 197)	Cholangitis (*n* = 14)	*P*	Non-pancreatitis (*n* = 190)	Pancreatitis (*n* = 21)	*P*
Pre-perforation									
No	174 (87.4%)	25 (12.5%)	0.003	189 (95%)	10 (5%)	0.004	184 (92.5%)	15 (7.5%)	0.000
Yes	6 (50%)	6 (50%)		8 (66.7%)	4 (33.3%)		6 (50%)	6 (50%)	
Pre-cholangitis									
No	150 (90.9%)	15 (9.1%)	0.000	162 (98.2%)	3 (1.8%)	0.000	160 (97%)	5 (3%)	0.000
Yes	30 (65.2%)	16 (34.8%)		35 (76.1%)	11 (23.9%)		30 (65.2%)	16 (34.8%)	
Type									
Non-4A	141 (92.8%)	11 (7.2%)	0.000	150 (98.7%)	2 (1.3%)	0.000	146 (96.1%)	6 (3.9%)	0.000
4A	39 (66.1%)	20 (33.9%)		47 (79.7%)	12 (20.3%)		44 (74.6%)	15 (25.4%)	
Gender									
Female	134 (85.4%)	23 (14.6%)	0.976	147 (93.6%)	10 (6.4%)	0.758	143 (91.1%)	14 (8.9%)	0.392
Male	46 (85.2%)	8 (14.8%)		50 (92.6%)	4 (7.4%)		47 (87%)	7 (13%)	
Blood transfusion									
No	139 (86.3%)	22 (13.7%)	0.449	153 (95%)	8 (5%)	0.103	146 (90.7%)	15 (9.3%)	0.592
Yes	41 (82%)	9 (18%)		44 (88%)	6 (12%)		44 (88%)	6 (12%)	
Shape of the cyst									
Cystic	112 (86.2%)	18 (13.8%)	0.66	121 (93.1%)	9 (6.9%)	0.831	117 (90%)	13 (10%)	0.977
Non-cystic	68 (84%)	13 (16%)		76 (93.8%)	5 (6.2%)		73 (90.1%)	8 (9.9%)	
Anaemia									
No	150 (89.8%)	17 (10.2%)	0.001	163 (97.6%)	4 (2.4%)	0.000	156 (93.4%)	11 (6.6%)	0.003
Yes	30 (68.2%)	14 (31.8%)		34 (77.3%)	10 (22.7%)		34 (77.3%)	10 (22.7)	
Removal drainage	9 (8,10)	13 (9,15)	0.000	9 (8,10)	13.5 (11.5,15)	0.000	9 (8,10)	13 (9,15)	0.000
Duration of surgery	215 (191,250)	235 (215,280)	0.001	215 (191,250)	225 (213.75,276.25)	0.223	220 (195,250)	235 (217.5,277.5)	0.005
Blood loss	10 (8,20)	10 (8,20)	0.921	10 (8,20)	10 (8,20)	0.543	10 (8,20)	10 (9,30)	0.413
Duration of hospital stay	20 (17,24)	21 (18,22)	0.824	20 (17,24)	20.5 (17.75,23)	0.817	21 (17,24)	20 (17.5,21.5)	0.446
Postoperative hospital stay	11 (10,13)	12 (10,14)	0.735	11 (10,13)	11.5 (9,13)	0.590	12 (10,13)	11 (10,13)	0.211
Cyst diameter	5 (4,8)	7 (6,8.9)	0.001	5 (4,8)	7.9 (6.6,10.13)	0.007	5 (4,8)	8 (6.4,10.25)	0.000
WBC	8.45 (6.47,10.42)	8.32 (5.9,11.89)	0.744	8.45 (6.47,10.42)	8.00 (6.91,11.89)	0.996	8.43 (6.44,10.42)	8.4 (6.545,13.51)	0.632
Age	26 (13.5,55.75)	27 (9,79)	0.555	26 (13.5,55.75)	22.5 (2.18,63.75)	0.636	26.5 (14.5,56.25)	27 (4.25,70)	0.935
Weight	12 (9.81,18)	13.5 (8,25)	0.576	12 (9.81,18)	12.75 (4.65,20.63)	0.930	12 (9.9375,18.5)	13.5 (5.7,24.5)	0.809
PLT	362.5 (299.5,447)	346 (282,391)	0.255	362.5 (299.5,447)	326 (289.25,384.25)	0.233	364.5 (301,447)	336 (236,395)	0.087
ALT	37 (15,85)	53 (22,83)	0.342	37 (15,85)	45 (16,90.5)	0.897	38.5 (15,88.25)	53 (21.5,65.5)	0.760
AST	38.5 (26,70.25)	56 (24,87)	0.552	38.5 (26,70.25)	44 (22.5,108.25)	0.995	39 (26,77.25)	48 (23.5,67.5)	0.802
GGT	160.5 (56,333)	623 (188,1090)	0.000	160.5 (56,333)	241.5 (177.75,938.75)	0.030	162 (56,336.75)	699 (241.5,1214)	0.000
APRI	0.28 (0.17,0.53)	0.36 (0.21,0.56)	0.284	0.28 (0.17,0.53)	0.37 (0.16,0.62)	0.749	0.285 (0.17,0.56)	0.36 (0.205,0.435)	0.586
AKP	236 (178.25,352)	366 (301,466)	0.000	236 (178.25,352)	391 (203,480)	0.104	241 (179,388)	356 (281,461)	0.004
TBil	11.3 (5.65,32.1)	15.3 (8.7,37)	0.257	11.3 (5.65,32.1)	12.85 (6.58,20.58)	0.767	11.7 (5.8,32.45)	13.7 (6.35,34.85)	0.791
DBil	5.6 (2.3,20.25)	7.6 (3.4,15.6)	0.393	5.6 (2.3,20.25)	6.15 (3.1,10.58)	0.879	5.95 (2.3,20.475)	5.6 (3.3,10.85)	0.871
Total protein	62.35 (58.2,67)	60.2 (57.6,65.2)	0.202	62.35 (58.2,67)	59.75 (54.65,64.83)	0.190	62.35 (58.15,67)	60.2 (57.6,62.15)	0.079
Albumin	42.1 (39.13,45.48)	40.8 (35.9,43.8)	0.023	42.1 (39.13,45.48)	36.35 (33.65,38.43)	0.000	42.1 (39.075,44.95)	38.8 (35.6,43.65)	0.011
Amylase	42 (30.75,79)	256(37,653)	0.000	42(30.75,79)	220(25.5,558)	0.038	41(33.75,79.5)	356(126,664)	0.000
Lipase	30.5(21,54)	249(39,553)	0.000	30.5(21,54)	229.5(37,415.25)	0.002	31(21,58.75)	289(107,760)	0.000

### 3.2 Variable selection

**Figure 1:**
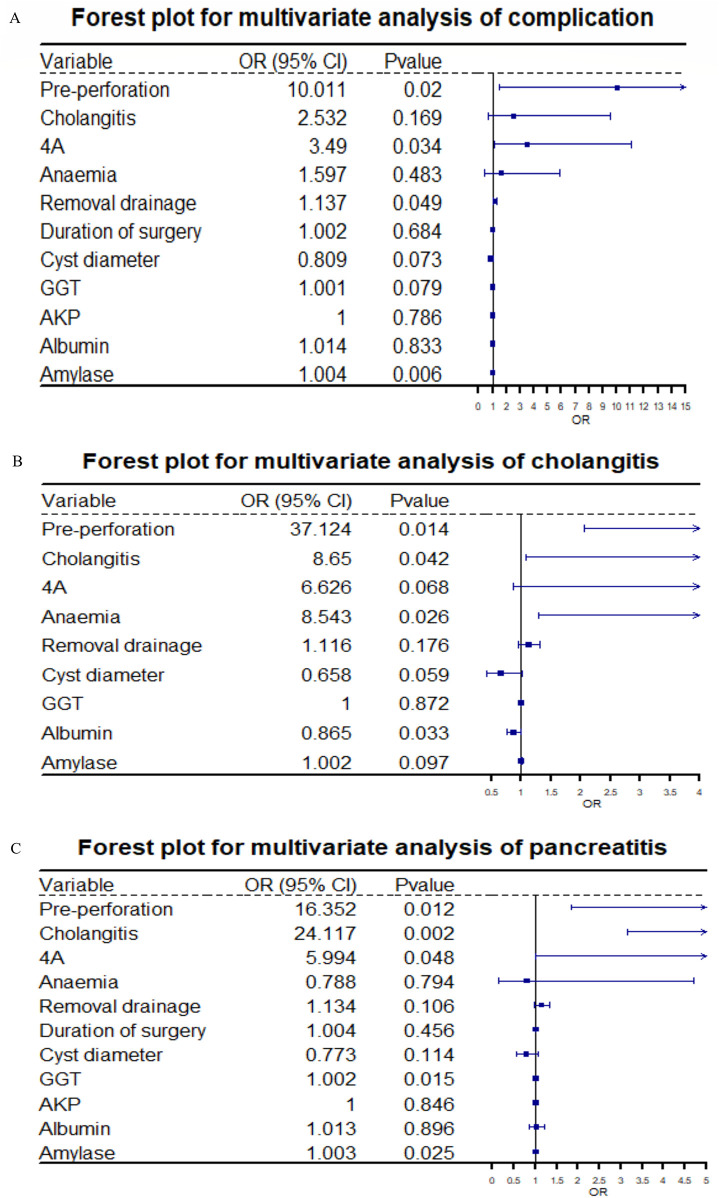
Panels **A, B**, and **C** respectively present the forest plots of multivariate logistic regression analysis results for total complications, cholangitis, and pancreatitis. On the far left of the figures are the variables included in the regression analysis; to the right of each variable are the corresponding results of the multivariate analysis, namely the odds ratio (OR) values and *P*-values.

Multivariate analysis showed an association between pre-perforation (*P* = 0.02), type 4A (*P* = 0.034), removal drainage (*P* = 0.049), and serum amylase (*P* = 0.006) with the development of postoperative complications. Pre-perforation (*P* = 0.014), pre-cholangitis (*P* = 0.042), anemia (*P* = 0.026), and serum albumin (*P* = 0.033) were associated with post-cholangitis. Pre-perforation (*P* = 0.012), pre-cholangitis (*P* = 0.002), type 4A (*P* = 0.048), GGT (*P* = 0.015), and amylase (*P* = 0.025) were associated with post-pancreatitis. Patients with pre-perforation had a significantly higher incidence of overall postoperative complications, including cholangitis and pancreatitis. Patients with preoperative cholangitis had a significantly higher incidence of postoperative pancreatitis and cholangitis. Type 4A classification was significantly associated with an increased incidence of postoperative complications, particularly pancreatitis. Details are shown in Figure 1.

**Figure 2:**
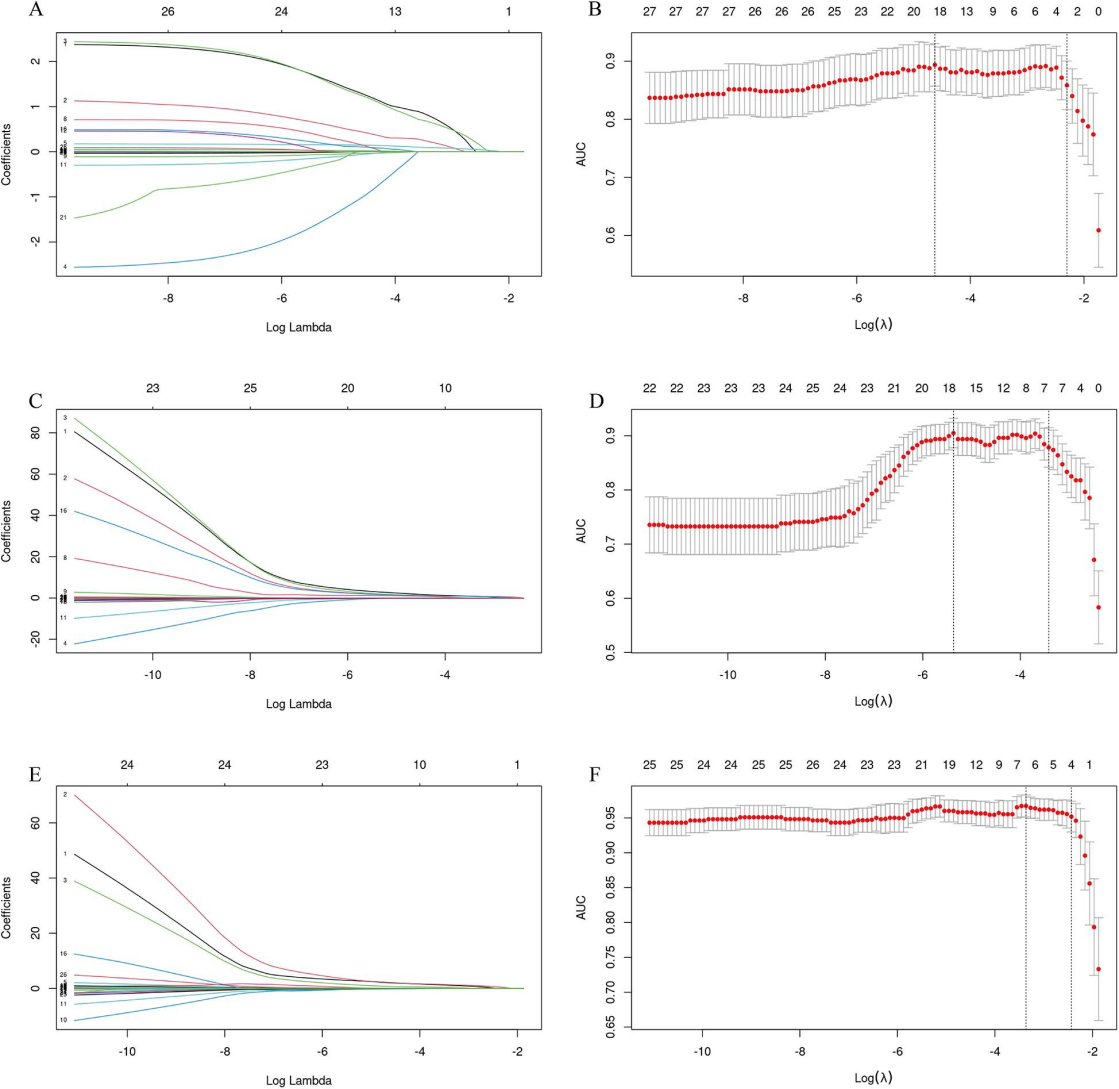
LASSO regression analysis was used to select risk factors. **(A,C,E)** The process of selecting the optimal value of the parameter *λ* in the Lasso regression model of complications, cholangitis and pancreatitis by the cross-validation method. The vertical dashed lines indicate the *λ* value corresponding to the minimum cross-validation error (left) and the *λ* value increased by one standard error (right). The minimum standard of complications includes removal drainage, GGT, amylase. Predictive minimum criteria for cholangitis includes pre-perforation, pre-cholangitis, type 4A, removal drainage, anemia, albumin, amylase. The minimum criteria for prediction of pancreatitis includes pre-perforation, pre-cholangitis, GGT, amylase. **(B,D,F)** The variable selection path of LASSO regression for complications, cholangitis and pancreatitis based on the optimal log *λ*, along with the corresponding coefficient changes at different *λ* values. Each colored line represents the coefficient of each feature.

A Lasso regression analysis was conducted on all variables. The results showed that the independent variables for complications, cholangitis, and pancreatitis decreased from 27 to 3 (removal drainage, GGT, amylase), 7 (pre-perforation, pre-cholangitis, type 4A, removal drainage, anemia, albumin, amylase), and 4 (pre-perforation, pre-cholangitis, GGT, amylase), respectively (Figure 2).

Model construction was conducted using variables selected through lasso regression and multivariate analysis separately. The collinearity was assessed using the variance inflation factor (VIF). VIF > 5 was considered indicative of severe multicollinearity between variables. The results confirmed the lack of collinearity among the selected variables (Supplementary Tables S1–S3).

### 3.3 Model construction and comparison

We used seven ML algorithms to construct predictive models for the three outcomes. Multivariate-logistics and multivariate-neural networks had the highest area under the ROC curve for predicting complications.

The variables screened by Lasso regression for predicting cholangitis (seven variables) performed significantly better than the multivariate analysis. KNN had the smallest area under the ROC curve in the prediction model. All other models showed excellent performance.

The performance of the models constructed from the factors screened by multivariate analysis and lasso regression did not differ significantly, and we selected the model with fewer factors included (four factors) for convenience. LR, SVM, and RF performed better in predicting pancreatitis (Table 2).

**Table 2:** Results of machine learning.

Predictive models		LR	KNN	SVM	CART	RF	XGB	NN
Complications-3								
	AUC	0.770	0.739	0.768	0.602	0.795	0.775	0.766
	Accuracy	0.875	0.141	0.828	0.875	0.875	0.891	0.844
	Precision	0.444	1.000	0.444	0.222	0.556	0.333	0.444
	Recall	0.571	0.141	0.400	0.667	0.556	0.750	0.444
	F1 score	0.500	0.247	0.421	0.333	0.556	0.462	0.444
Complications-4								
	AUC	0.788	0.669	0.669	0.611	0.785	0.745	0.778
	Accuracy	0.844	0.141	0.797	0.891	0.875	0.875	0.844
	Precision	0.556	1.000	0.222	0.222	0.556	0.556	0.667
	Recall	0.455	0.141	0.250	1.000	0.556	0.556	0.462
	F1 score	0.500	0.247	0.235	0.364	0.556	0.556	0.545
Cholangitis-4								
	AUC	0.840	0.806	0.852	0.832	0.891	0.895	0.840
	Accuracy	0.821	0.218	0.795	0.782	0.821	0.808	0.821
	Precision	0.765	1.000	0.706	0.588	0.765	0.824	0.765
	Recall	0.565	0.218	0.522	0.500	0.565	0.538	0.565
	F1 score	0.650	0.358	0.600	0.541	0.650	0.651	0.650
Cholangitis-7								
	AUC	0.905	0.557	0.902	0.955	0.953	0.943	0.926
	Accuracy	0.846	0.218	0.795	0.897	0.923	0.897	0.859
	Precision	0.765	1.000	0.765	1.000	1.000	1.000	1.000
	Recall	0.619	0.218	0.520	0.680	0.739	0.680	0.607
	F1 score	0.684	0.358	0.619	0.810	0.850	0.810	0.756
Pancreatitis-4								
	AUC	0.922	0.509	0.931	0.879	0.949	0.909	0.909
	Accuracy	0.854	0.317	0.829	0.793	0.890	0.878	0.829
	Precision	0.923	1.000	0.808	0.808	1.000	1.000	1.000
	Recall	0.706	0.317	0.700	0.636	0.743	0.722	0.650
	F1 score	0.800	0.481	0.750	0.712	0.852	0.839	0.788
Pancreatitis-5								
	AUC	0.912	0.527	0.930	0.875	0.964	0.913	0.903
	Accuracy	0.841	0.317	0.854	0.793	0.915	0.878	0.927
	Precision	0.885	1.000	0.923	0.846	1.000	1.000	0.923
	Recall	0.697	0.317	0.706	0.629	0.788	0.722	0.857
	F1 score	0.780	0.481	0.800	0.721	0.881	0.839	0.889

To better interpret and apply the model, we used LR to predict the three outcomes and develop nomograms.

### 3.4 Development and verification of nomograms

**Figure 3:**
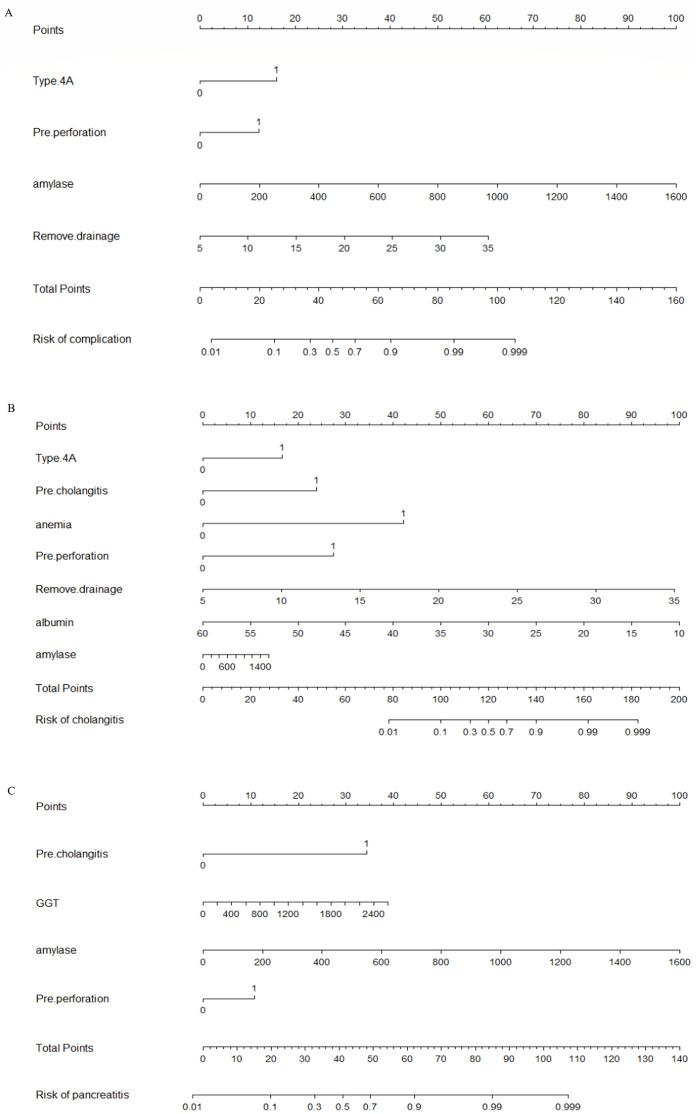
Nomogram for predicting postoperative complications in CBD. **(A)** Nomogram for predicting postoperative complications in CBD. The left column displays the scoring bar (at the top) and four parameters, each of which is scored with a vertical line to the scoring bar based on different parameter values. The total score is calculated, and a vertical line is drawn from the total score bar down to the “Risk of complications” section to obtain the probability of complications for the patient. **(B)** The nomogram for predicting postoperative cholangitis following CBD procedures. The leftmost column displays a scoring axis (top section) alongside seven predictive parameters. Each parameter is assigned a corresponding score via a vertical alignment to the top scoring bar, determined by its specific value. After calculating the total score, a vertical line is projected downward from the total score axis to the “Risk of cholangitis section”, thereby determining the patient's individualized probability of developing cholangitis. **(C)** Nomogram for predicting postoperative pancreatitis following CBD surgery. The leftmost column displays the scoring axis (top section) along with four predictive parameters. Each parameter connects vertically to the top scoring axis, with its corresponding score determined by its specific value. After calculating the total score, a vertical line is drawn downward from the total score axis to the “Risk of pancreatitis” area, thereby determining the individualized probability of the patient developing pancreatitis.

**Figure 4:**
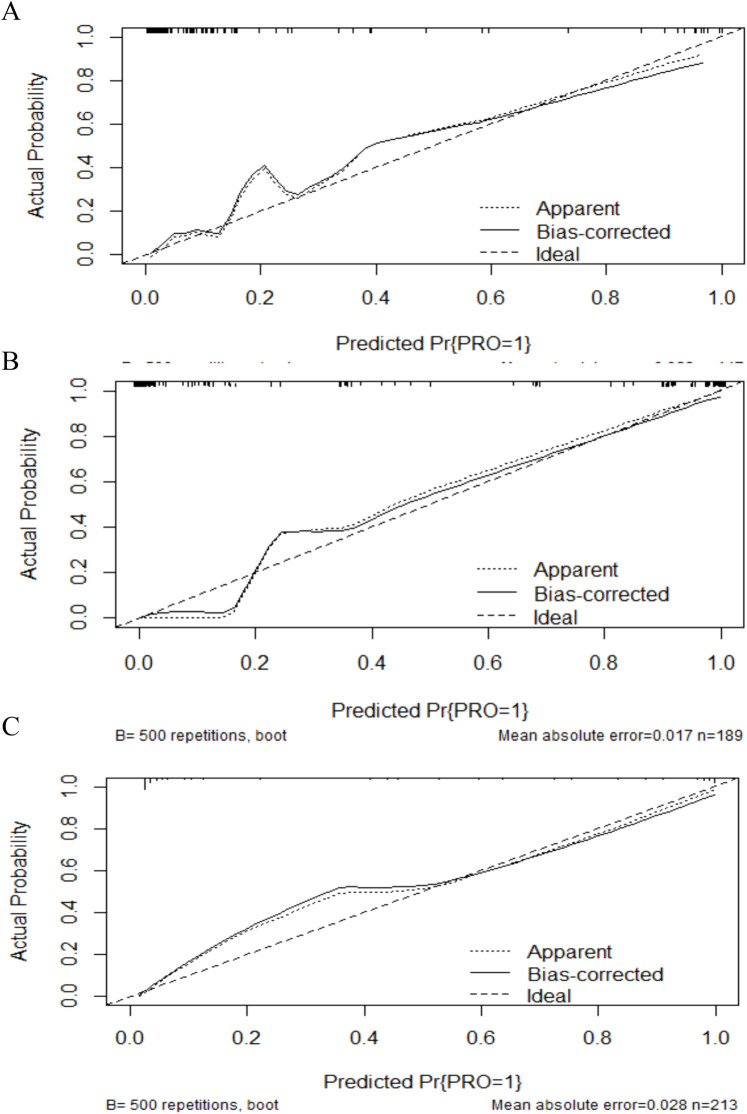
Calibration plot for predicting postoperative complications in CBD. **(A)** The calibration plot analysis result of complications. **(B)** The calibration plot analysis result of cholangitis. **(C)** The calibration plot analysis result of pancreatitis. The *Y*-axis scale represents the actual probability of complications, while the *X*-axis scale displays the predicted values calculated using the model. The dashed line indicates the fit between the original predicted values and the actual values, whereas the solid line represents the relationship between the calibrated predicted values and the actual values. Our predicted values show a high consistency with the observed values.

**Figure 5:**
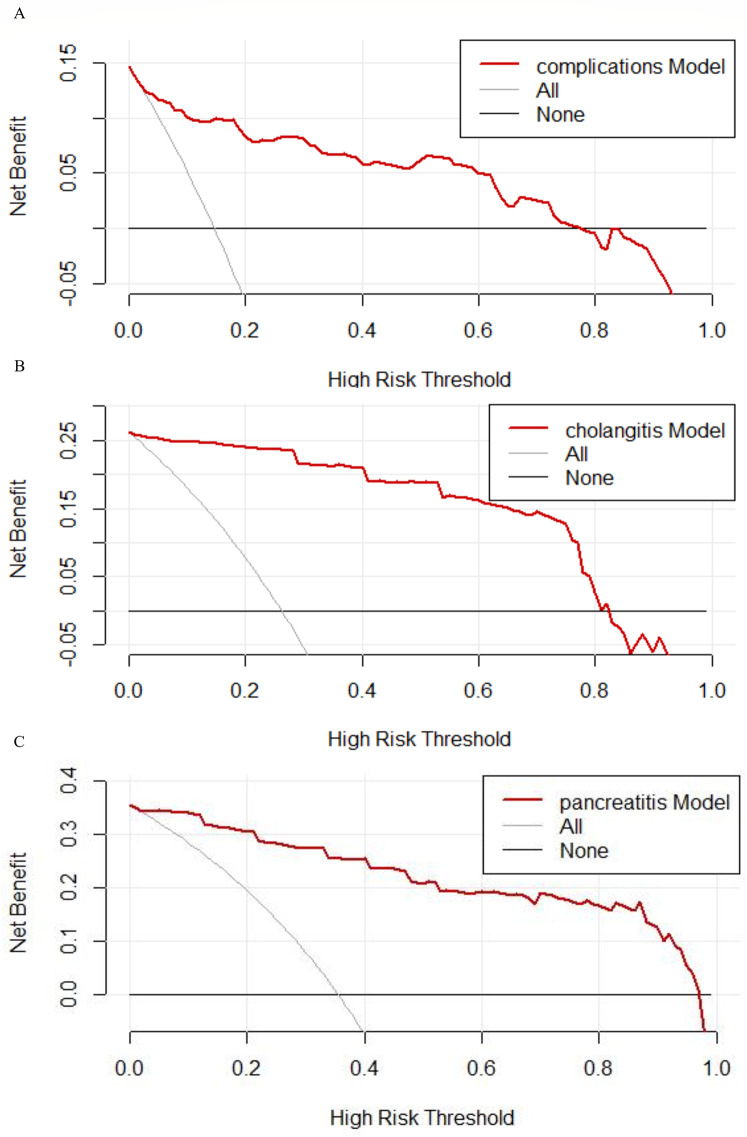
Decision curve for predicting postoperative complications in CBD. **(A)** The decision curve analysis result of complications. **(B)** The decision curve analysis result of cholangitis. **(C)** The decision curve analysis result of pancreatitis. The net benefit is calculated at different threshold probabilities. The black curve represents the scenario in which no interventions are implemented, while the gray curve represents the scenario in which interventions are applied to all patients.

Nomograms were developed to predict complications of cholangitis and pancreatitis, based on the screened independent risk factors (Figure 3). The calibration curves showed a high degree of consistency between the predicted and observed probabilities, demonstrating the high accuracy of the predictive models (Figure 4). Decision curve analysis was used to facilitate decision-making when evaluating the clinical applicability (Figure 5).

## 4 Discussion

In this study, we analyzed the clinical data of 211 patients and used seven ML algorithms to develop predictive models for complications, cholangitis, and pancreatitis after CBD surgery. By comparison, LR showed superior clinical predictive values, with AUCs of 0.788, 0.905, and 0.922 for the prediction of complications, cholangitis, and pancreatitis, respectively, in the internal validation dataset. Additionally, we developed nomograms to facilitate the clinical application of the models.

CBD is a common structural abnormality of the bile ducts that may lead to complications such as bile duct stones, pancreatitis, biliary tract infections, and even bile duct cancer. The overall complication rate may reach 60%, while the cancer incidence may be as high as 26% in patients aged over 40 (12, 13). Therefore, research on the complications of CBD is imperative.

Our analysis revealed that preoperative perforation is a significant risk factor for postoperative complications, cholangitis, and pancreatitis. This finding highlights the critical warning value of preoperative perforation, indicating a greater severity of the disease. Notably, this association has not been highlighted in previous studies. The underlying mechanism may involve persistent local chronic inflammation following perforation, leading to significant adhesion formation (14). In previous studies, patients with perforations were younger and had significantly higher levels of GGT and C-reactive protein (CRP) (15–17). Patients with perforations also had higher WBCs and lower albumin levels (18). In our study, the incidence of complications and pancreatitis was higher in type-4A category, which is consistent with previous findings (19, 20). Extended duration of postoperative drainage days has been found to be associated with the development of postoperative complications. This may be attributable to the fact that prolonged time to drainage implies more localized exudation and severe inflammation.

Based on our study, patients identified as high-risk by the model (e.g., those with preoperative biliary perforation, Todani type 4A, or significant elevation of serum amylase) should undergo preoperative interventions. Specifically, modifiable factors (e.g., hypoalbuminemia, anemia) should be addressed through nutritional support—such as albumin supplementation and iron therapy—to enhance tissue healing capacity. Postoperatively, patients at high risk should be closely monitored for changes in the characteristics of drainage fluid, and individualized follow-up plans should also be established (e.g., closer follow-up and monitoring). Furthermore, priority should be given to early warning and intervention for severe complications. For example, in patients at high risk of cholangitis, prophylactic antibiotics should be administered postoperatively, and potential abnormalities (e.g., biliary stones) should be promptly addressed.

Some limitations of this study should be acknowledged. 1) This was a single-centre study with no external data validation. 2) Due to the limited follow-up duration, we did not find bile duct cancer during the follow-up period, which precluded prediction of bile duct cancers. We will continue to conduct longer follow-ups to obtain data on bile duct cancers. 3) The number of patients who experienced complications was limited, which remains a study limitation. Despite these shortcomings, there are many strengths of our study. This study is the first predictive model about postoperative complications, cholangitis, and pancreatitis after surgery for CBD. Additionally, we developed nomograms for clinical application.

## 5 Conclusion

We developed and validated a prediction model for postoperative complications of cholangitis and pancreatitis after surgery for CBD. Clinical application of the model can help prevent postoperative complications of CBD.

## Data Availability

Publicly available datasets were analyzed in this study. This data can be found here: https://data.mendeley.com/datasets/hxhkgwv2pv/1.

## References

[1] Wang SE Chen SC Shyr BU Shyr YM. Robotic assisted excision of type I choledochal cyst with Roux-en-y hepaticojejunostomy reconstruction. Hepatobiliary Surg Nutr. (2017) 6(6):397–400. 10.21037/hbsn.2017.01.15 29312974 PMC5756759

[2] Hamada Y Ando H Kamisawa T Itoi T Urushihara N Koshinaga T Diagnostic criteria for congenital biliary dilatation 2015. J Hepatobiliary Pancreat Sci. (2016) 23(6):342–6. 10.1002/jhbp.346 26996969

[3] Kamisawa T Kaneko K Itoi T Ando H. Pancreaticobiliary maljunction and congenital biliary dilatation. Lancet Gastroenterol Hepatol. (2017) 2(8):610–8. 10.1016/s2468-1253(17)30002-x 28691687

[4] Kim NY Chang EY Hong YJ Park S Kim HY Bai SJ Retrospective assessment of the validity of robotic surgery in comparison to open surgery for pediatric choledochal cyst. Yonsei Med J. (2015) 56(3):737–43. 10.3349/ymj.2015.56.3.737 25837180 PMC4397444

[5] Jang JY Yoon YS Kang MJ Kwon W Park JW Chang YR Laparoscopic excision of a choledochal cyst in 82 consecutive patients. Surg Endosc. (2013) 27(5):1648–52. 10.1007/s00464-012-2646-0 23239299

[6] Woo R Le D Albanese CT Kim SS. Robot-assisted laparoscopic resection of a type I choledochal cyst in a child. J Laparoendosc Adv Surg Tech A. (2006) 16(2):179–83. 10.1089/lap.2006.16.179 16646713

[7] Metcalfe MS Wemyss-Holden SA Maddern GJ. Management dilemmas with choledochal cysts. Arch Surg. (2003) 138(3):333–9. 10.1001/archsurg.138.3.333 12611583

[8] Fujishiro J Masumoto K Urita Y Shinkai T Gotoh C. Pancreatic complications in pediatric choledochal cysts. J Pediatr Surg. (2013) 48(9):1897–902. 10.1016/j.jpedsurg.2012.12.038 24074664

[9] Tanaka Y Tainaka T Hinoki A Shirota C Sumida W Yokota K Risk factors and outcomes of bile leak after laparoscopic surgery for congenital biliary dilatation. Pediatr Surg Int. (2021) 37(2):235–40. 10.1007/s00383-020-04791-0 33392697

[10] von Elm E Altman DG Egger M Pocock SJ Gøtzsche PC Vandenbroucke JP. The strengthening the reporting of observational studies in epidemiology (STROBE) statement: guidelines for reporting observational studies. Lancet. (2007) 370(9596):1453–7. 10.1016/s0140-6736(07)61602-x 18064739

[11] Todani T Watanabe Y Narusue M Tabuchi K Okajima K. Congenital bile duct cysts: classification, operative procedures, and review of thirty-seven cases including cancer arising from choledochal cyst. Am J Surg. (1977) 134(2):263–9. 10.1016/0002-9610(77)90359-2 889044

[12] Dou J Jiang N Zeng J Wang S Tian S Shan S Novel 3D morphological characteristics for congenital biliary dilatation diagnosis: a case-control study. Int J Surg. (2024) 110(5):2614–24. 10.1097/js9.0000000000001204 38376858 PMC11093423

[13] Ishibashi H Shimada M Kamisawa T Fujii H Hamada Y Kubota M Japanese Clinical practice guidelines for congenital biliary dilatation. J Hepatobiliary Pancreat Sci. (2017) 24(1):1–16. 10.1002/jhbp.415 28111910

[14] Kamisawa T Ando H Suyama M Shimada M Morine Y Shimada H. Japanese Clinical practice guidelines for pancreaticobiliary maljunction. J Gastroenterol. (2012) 47(7):731–59. 10.1007/s00535-012-0611-2 22722902

[15] Zhang G Wang H Hu J Yang C Tan B Hu J A nomogram for predicting choledochal cyst with perforaation. Pediatr Surg Int. (2024) 40(1):129. 10.1007/s00383-024-05710-3 38727920 PMC11087341

[16] Fukuzawa H Urushihara N Miyakoshi C Kajihara K Kawahara I Isono K Clinical features and risk factors of bile duct perforation associated with pediatric congenital biliary dilatation. Pediatr Surg Int. (2018) 34(10):1079–86. 10.1007/s00383-018-4321-6 30076449

[17] Zhang S Cai D Chen Q Zhang Y Chen K Jin Y Value of serum GGT level in the timing of diagnosis of choledochal cyst perforation. Front Pediatr. (2022) 10:921853. 10.3389/fped.2022.921853 36046482 PMC9421046

[18] Kim YJ Kim SH Yoo SY Kim JH Jung SM Lee S Comparison of clinical and radiologic findings between perforated and non-perforated choledochal cysts in children. Korean J Radiol. (2022) 23(2):271–9. 10.3348/kjr.2021.0169 35029072 PMC8814706

[19] Tanaka R Nakamura H Yoshimoto S Okunobo T Satake R Doi T. Postoperative anastomotic stricture following excision of choledochal cyst: a systematic review and meta-analysis. Pediatr Surg Int. (2022) 39(1):30. 10.1007/s00383-022-05293-x 36454303

[20] Kim JH Choi TY Han JH Yoo BM Kim JH Hong J Risk factors of postoperative anastomotic stricture after excision of choledochal cysts with hepaticojejunostomy. J Gastrointest Surg. (2008) 12(5):822–8. 10.1007/s11605-007-0415-5 18058186

