# Supplementary material for: Nomograms for postoperative complications in congenital biliary dilatation: a retrospective cohort study
Source: Front Pediatr. 2025 Oct 28;13:1654592. doi: 10.3389/fped.2025.1654592 (PMC12602394; doi:10.3389/fped.2025.1654592)
Supplement: Supplementary file 1 [file Datasheet1.docx]

Supplementary Table

| Supplementary table1 VIF test results of complications. | | | | |
| --- | --- | --- | --- | --- |
|  | Pre-perforation | Type 4A | Remove drainage | amylase |
| Pre-perforation | 1.000 | 0.075 | 0.134 | 0.062 |
| Type 4A | 0.075 | 1.000 | 0.279 | 0.273 |
| Remove drainage | 0.134 | 0.279 | 1.000 | 0.325 |
| amylase | 0.062 | 0.273 | 0.325 | 1.000 |

| Supplementary table2 VIF test results of cholangitis. | | | | | | | |
| --- | --- | --- | --- | --- | --- | --- | --- |
|  | Pre-perforation | Pre-cholangitis | Type 4A | Remove drainage | anemia | albumin | amylase |
| Pre-perforation | 1.000 | 0.351 | 0.144 | 0.178 | 0.058 | -0.256 | 0.040 |
| Pre-cholangitis | 0.351 | 1.000 | 0.452 | 0.341 | 0.302 | -0.398 | 0.375 |
| Type4A | 0.144 | 0.452 | 1.000 | 0.361 | 0.417 | -0.335 | 0.308 |
| Remove drainage | 0.178 | 0.341 | 0.361 | 1.000 | 0.301 | -0.139 | 0.334 |
| anemia | 0.058 | 0.302 | 0.417 | 0.301 | 1.000 | -0.282 | 0.170 |
| albumin | -0.256 | -0.398 | -0.335 | -0.139 | -0.282 | 1.000 | -0.229 |
| amylase | 0.040 | 0.375 | 0.308 | 0.334 | 0.170 | -0.229 | 1.000 |

| Supplementary table3 VIF test results of pancreatitis. | | | | |
| --- | --- | --- | --- | --- |
|  | Pre-perforation | Pre-cholangitis | GGT | amylase |
| Pre-perforation | 1.000 | 0.291 | 0.243 | 0.049 |
| Pre-cholangitis | 0.291 | 1.000 | 0.213 | 0.333 |
| GGT | 0.243 | 0.213 | 1.000 | 0.407 |
| amylase | 0.049 | 0.333 | 0.407 | 1.000 |
